# Biothreat Reduction and Economic Development: The Case of Animal Husbandry in Central Asia

**DOI:** 10.3389/fpubh.2015.00270

**Published:** 2015-12-23

**Authors:** Robert Walker, Jason Blackburn

**Affiliations:** ^1^Department of Geography, Center for Latin American Studies, University of Florida, Gainesville, FL, USA; ^2^Department of Geography, Emerging Pathogens Institute, University of Florida, Gainesville, FL, USA

**Keywords:** central Asia, biothreats, economic development, conservation of natural resources, geospatial analysis

## Abstract

Improving human welfare is a critical global concern, but not always easy to achieve. Complications in this regard have been faced by the states of the Former Soviet Union, where socialist-style economic institutions have disappeared, and the transition to a market economy has been slow in coming. Lack of capital, ethnic conflict, and political instability have at times undermined the institutional reform that would be necessary to enable economic efficiency and development. Nowhere are such challenges more pronounced than in the new nation states of central Asia, including Kazakhstan, Kyrgyzstan, Tajikistan, Turkmenistan, and Uzbekistan. Here, a severe climate limits agriculture, and industrialization has been inhibited by lack of infrastructure, low levels of human capital, and a scarcity of financial resources. These conditions are aggravated by the fact that the central Asian states are landlocked, far from centers of market demand and capital availability. Despite these daunting barriers, development potential does exist, and the goal of the paper is to consider central Asia’s pastoral economy, with a focus on Kazakhstan, which stands poised to become a regional growth pole. The article pursues its goal as follows. It first addresses the biothreat situation to central Asian livestock herds, the most significant existing impediment to realizing the full market potential of the region’s animal products. Next, it provides an outline of interventions that can reduce risk levels for key biothreats impacting central Asia, namely foot and mouth disease (FMD), which greatly impacts livestock and prohibits export, and Brucellosis, a bacterial zoonosis with high incidence in both humans and livestock in the region. Included is an important success story involving the FMD eradication programs in Brazil, which enabled an export boom in beef. After this comes a description of the epidemiological situation in Kazakhstan; here, the article considers the role of wildlife in acting as a possible disease reservoir, which presents a conservation issue for the Kazakhstani case. This is followed by a discussion of the role of science in threat reduction, particularly with respect to the potential offered by geospatial technologies to improve our epidemiological knowledge base. The article concludes with an assessment of the research that would be necessary to identify feasible pathways to develop the economic potential of central Asian livestock production as changes in policy are implemented and livestock health improves.

## Introduction

Improving human welfare is a critical global concern, but not always easy to achieve. Complications in this regard have been faced by the states of the Former Soviet Union (FSU), where socialist-style economic institutions have disappeared, and the transition to a market economy has been slow in coming. Lack of capital, ethnic conflict, and political instability have at times undermined the institutional reforms that would be necessary for spurring economic efficiency and development. Nowhere are such challenges more pronounced than for the 64,000,000 people who reside in Central Asia, which includes the new nation states of Kazakhstan, Kyrgyzstan, Tajikistan, Turkmenistan, and Uzbekistan (1). Here, a severe climate limits agriculture, and industrialization has been inhibited by lack of infrastructure, low levels of human capital, and a scarcity of financial resources. These conditions are aggravated by the fact that the region is landlocked, far from centers of market demand and capital availability. Despite these daunting barriers, development potential does exist, and the goal of the paper is to consider Central Asia’s pastoral economy, with a focus on Kazakhstan, whose ~18 million inhabitants are distributed over an expanse of 2,724,900 km^2^, making it one of the world’s largest countries.

Kazakhstan’s economy has grown dramatically in recent years, lifting it to the status of a middle income country, with per-capita gross domestic product (GDP) at 11,550 US$. In that these gains are in large part based on the extraction of fossil fuels, the Kazakhstani government seeks to diversify its economic development strategy with an eye toward expanding agricultural exports, particularly beef (2). This reflects genuine opportunity, given global market expansion due to rising incomes and changing consumption patterns (3). Increasingly, middle income countries are finding ways to engage in the international trade of beef, despite continuing dominance by large producers, such as the US and Brazil (3). Extensive natural rangelands, together with a long-standing cultural tradition of animal husbandry, create a significant potential for Kazakhstan based on livestock management. That said, a number of issues constrain this potential at the present time, and it is the goal of the present paper to address one of them, namely, the regional biothreat situation.

The article pursues its goal as follows. It starts by describing the main biothreats presently affecting central Asian livestock herds, namely, foot and mouth disease (FMD) and Brucellosis. Like its central Asian neighbors, Kazakhstan’s cattle herds and small stock consisting of goats and sheep have faced periodic problems with FMD and Brucellosis, and both diseases remain under official government surveillance (4). After this, the article moves on to a discussion of policy interventions that have managed to control or eradicate FMD, whose outbreaks bring substantial economic losses to those engaged in international trade. Policy is considered in the context of a case study of Brazil, a country that has largely suppressed FMD and, as a consequence, emerged as a major beef exporter. Parallels with Kazakhstan make the Brazilian experience relevant to Kazakhstani development efforts. After addressing policy, the article considers the role of computational science in threat reduction through the analysis and prediction of outbreak patterns. The article concludes with an assessment of the research that would be necessary to identify feasible pathways to develop the economic potential of Kazakhstani livestock production.

## The Biothreat Situation in Central Asia

Livestock herds in many parts of the world are vulnerable to disease agents, some of which are capable of infecting humans. Two of the most significant with respect to economic impacts are FMD, a picornavirus of the genus *Apthovirus*, and Brucellosis, a bacterial zoonosis of the genus *Brucella*. FMD affect bovids and other hooved animals, both domestic and wild, causing fever and raising blisters (vesicules). Susceptible animals include cattle, pigs, sheep, goats, and camels. Although young cattle often die, mortality is low for mature animals, and impacts come from reduced outputs of animal-based products, such as milk and meat. Cattle and sheep are also susceptible to Brucellosis, a bacterial infection that can pass to the human population, where it is known variously as Malta Fever, Undulant Fever, etc. Human exposure typically occurs through the consumption of unpasteurized milk, and causes febrile symptoms, muscular pain, and sweating. Antibiotics have eliminated human mortality, which was never very high, although chronic sequelae (e.g., sacroiliitis, hepatic disease, endocarditis, and meningitis) can be serious. As for animal populations, *Brucella* causes economic damages by inducing spontaneous abortions, known in extreme cases as *abortion storms*. This obviously lowers reproductive potential and therefore rates of herd expansion, with commercial consequences. Effective vaccines have been produced for FMD (5), but efficacy is difficult to assess, and for Kazakhstan estimates do not exist. Moreover, the virus (FMDV) is capable of changing and possesses multiple serotypes, as discussed below. Thus, FMD prevention programs based on vaccination must remain vigilant to an evolving pathogen. As for Brucellosis in animals, a vaccine exists but prevention is best achieved by pasteurization of milk and cheese.

### Epidemiology of FMD and Brucellosis in Kazakhstan Today

We now consider the epidemiology of FMD and Brucellosis, as well as challenges to formulating eradication and control policy given Kazakhstan’s ecological context. This is followed by an overview of recent Kazakhstani history, which has significant implications for veterinary policy and practice.

#### Foot and Mouth Disease

**Figure 1:**
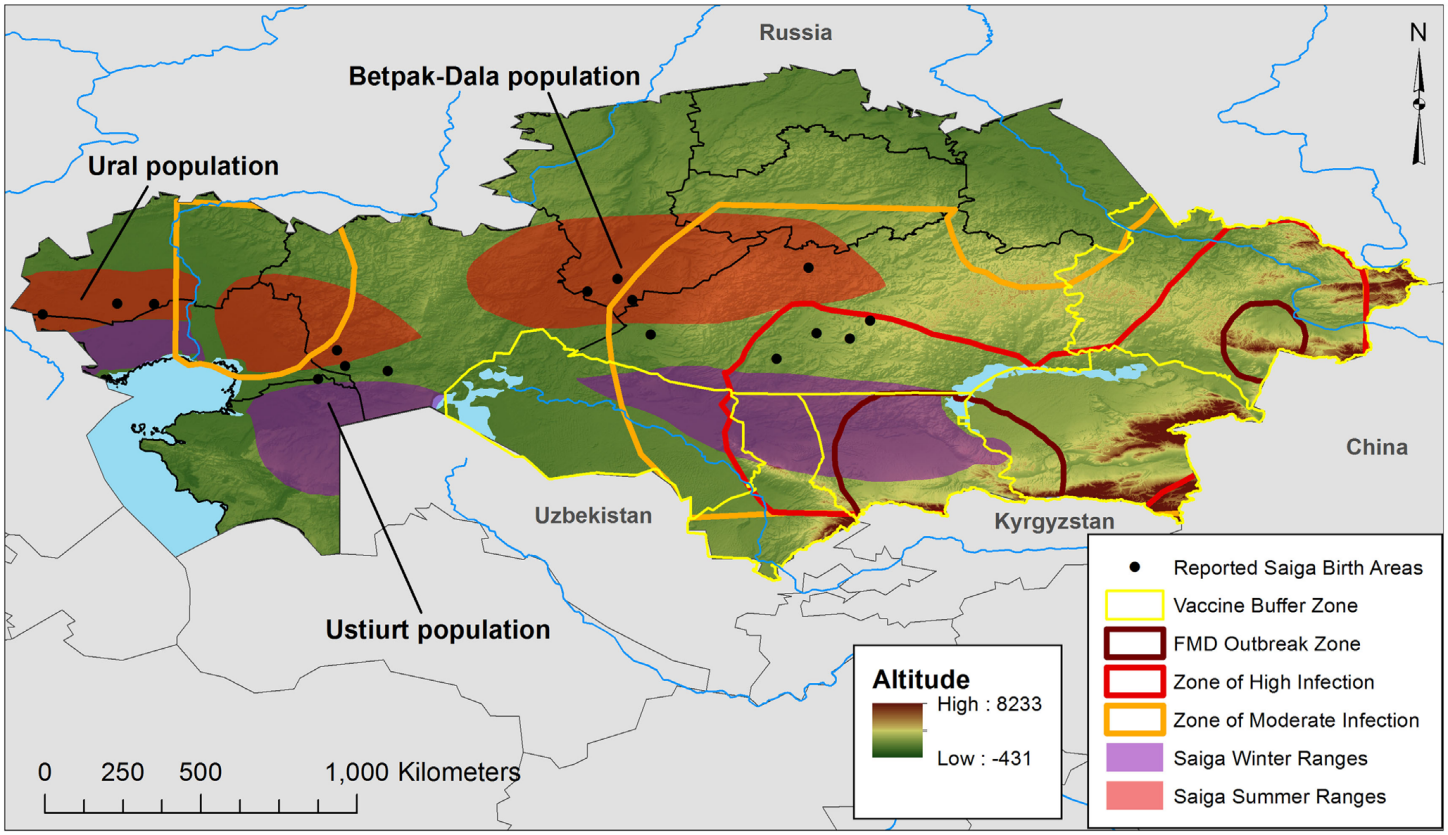
**The wildlife factor**. Map of Kazakhstan illustrating the recent FMD outbreak and risk zones and the current vaccination buffer, as defined by Sytnik et al. (11) after the 2011–2012 livestock FMD outbreaks. *Saiga* populations, birthing areas, and seasonal ranges were adapted from Bekenov et al. (56) and Fry (57) to illustrate areas where surveillance and disease control should consider wildlife populations and conservation issues.

Foot and mouth disease is caused by a positive, single-stranded RNA virus, which possesses seven known serotypes (O, A, Asia 1, C, SAT 1, SAT 2, and SAT 3), although serotype O is the most prevalent (6). The disease spreads easily, as infected animals shed the virus in secretions and excretions; transmission can be airborne, through contact with animal fluids, or via mechanical transmission of infected materials between properties (7). Overland airborne transmission can exceed 10 km, which makes FMD particularly difficult to contain (8). ELISA tests are capable of detecting the presence of antibodies to FMDV (test prevalence), and also of identifying false positives due to prior vaccination (4). Such procedures provide imperfect measures of FMD (and Brucellosis) infection, although test prevalence may be used to estimate actual prevalence via Bayesian techniques (9). Despite diagnostic limitations, the Kazak National Reference Veterinary Center (NRVC) has reported serotypes O, A, and the A22 subtype in Kazakhstan, with evidence of disease in cattle, small stock (sheep and goats), swine, and camels (10). Between 1955 and 2007, (test) seroprevalence of FMD in cattle was assessed nationally at 3.78% for type A, 4.3% for type O, and 2.72% for A22. For small stock, the respective rates were 8.86, 9.63, and 3.83%, and for swine, 6.77, 6.32, and 7.54. Only types A and O were reported in camels, at 0.96 and 7.11%, respectively (10). Geographically, virus type O shows wide distribution, with cattle and small-stock infections reported in nearly all Kazakh oblasts (state/province equivalents). A similarly wide range is documented for serotype A, with small-stock reports primarily concentrated in southern and north central oblasts. A post-soviet survey of nearly 1,000 animals in 1997–1998 (six oblasts) showed high FMD rates in cattle that ranged from 2.9 to 52.8%, with the highest rate found in southern Kazakhstan oblasts (4). Rates were lower in small stock in that survey (0–22.0%), with the highest prevalence in the Aktiubinsk oblast. Outbreaks of types A and O have been reported in 2011 and 2012. FMD appears to occur in all oblasts, with the greatest risk in southern and eastern Kazakhstan, along the Kyrgyz, China, and Russian borders, illustrating a significant trans-boundary risk of transmission (11, 12). Figure 1 illustrates the zones of risk according to NRVC in 2013, as defined by the concentration of cases.

#### Brucellosis

The newly formed Central Asian Republics represent one of the most significant loci of human Brucellosis infections in the world (13). With respect to Kazakhstan, the human incidence rose from 4.7 in 1967 to 15.3 cases per 100,000 in 1986, a direct consequence of FSU policy encouraging meat and dairy production (14). Rates remained high following the FSU’s collapse after 1991, with an incidence lingering at 11.6 per 100,000, in line with the Lundervold et al. (4) estimate of 10.8 per 100,000 in 1989. Grushina et al. (15) reported annual morbidity at 17.5 per 100,000, noting that the Almaty region exceeds the national rate with 22.9 cases per 100,000 in 2006. Almaty’s sub-districts Enbeksi-Kazakh and Zhambyl show very high rates, at 56.4 and 32.1, respectively (15). Pappas et al. (13) report that human *Brucella* infections in both Kazakhstan and Tajikistan are on the rise, on the basis of an evaluation of OIE and WHO reports. As with FMD, ELISA tests detect antibodies to *Brucella* in animals, and a serological survey (1997–1998) documented Brucellosis in cattle and small stock across eleven rayons (county/district equivalent) in south central Kazakhstan (4). Government statistics for small stock indicate seroprevalence (total sero-positive divided by the sample population) ranging from 0 to 1.1%, as compared to survey-specific rates of from 0 to 1.8% (4). Infection in neighboring countries may reach even higher levels, indicating a worrisome region-wide disease background. Seroprevalance in Tajikistan ranges from 0.53 to 6.96% at the rayon level (16), and clusters of sero-positivity in Armenian cattle and small stock show strong variation across time and space (17).

#### The Wildlife Factor

Kazakhstani rangelands cover 1.8 million km^2^, about 70% of the national territory, and a semi-arid climate (300 mm annual precipitation) produces forage capable of supporting large populations of ruminant herbivores, both domesticated and wild (18). It should come as no surprise then that serology has documented Brucellosis in both livestock and multiple taxa of Kazakh wildlife, including maral deer, mountain sheep, mountain goats, roe deer, and *Saiga* antelope (14). There is also historical evidence of FMD between 1955 and 1974. Outbreak control for either disease is especially complicated by the presence of large herds of *Saiga* antelope that group into three major populations – the Ural, Ustiurt, and Betpak-Dala – spread across thousands of kilometers of longitude. This east–west distribution is complemented by north–south migrations between winter and summer grazing lands. Given its range and mobility, the *Saiga* antelope could carry both pathogens over large parts of Kazakhstan, where it might easily spread. This is suggested by Figure 1, which illustrates the risk zones for infected livestock during recent outbreaks (2011–2012). As shown, the zones of moderate infection extend deep into Kazakhstan, in a manner suggesting that seasonal antelope movements play a role in FMD livestock outbreaks; likewise, infected livestock could spread the disease to Saiga, which raises conservation issues, given the antelope is recognized by the Convention on International Trade in Endangered Species (CITES). Further research is necessary to document the exact mechanism and extent of cross-species transmission.

#### Recent Historical Considerations

The biothreat situation in Kazakhstan today results from an interaction between environmental conditions and a turbulent history of absorption into the USSR, the trauma of a devastating famine that killed nearly 1.5 million people, and ethnic tensions resulting from the Soviet Union’s policy of distributing Russian nationals across the far reaches of its socialist empire (19). Prior to its incorporation into the USSR in 1930, the Kazaks practiced a mainly small-stock nomadism that accessed distinct ecological zones with two annual cycles. These comprise a latitudinal movement starting in the spring and covering 200–2000 km along a south–north axis, and an altitudinal one from the plains to the mountains during summer months (18). Sovietization refocused the Kazakhstani rural economy on cattle and wheat, and imported new agricultural institutions in the form of large state enterprises. Investments in veterinarian services were made, and agricultural activity in general became more capital intensive. That said, the state enterprises and the political nature of the USSR disrupted traditional forms of governance based on family clans (20).

Economic and social adjustments following the collapse of the USSR proved difficult, as was the case throughout Central Asia, with the physical constraints of its climate and geography. The Kazaks resumed old nomadic practices, but without the benefit of the social structures and institutions that had regulated transhumance and resource access for centuries (20, 21). It should come as no surprise that the early years of transition to a market economy brought widespread economic dislocation. The small-stock herd fell from 34.2 to 13.7 million animals between 1993 and 1996 (4). Similarly, the cattle herd of 7 million animals in 1995 fell to 4 million by 2000, with a slow recovery to 6 million animals by 2010 (22). From 1995 to 2010, the share of beef cattle in the national herd fell from 4 to 1%, and slaughter weights declined to 299 kg, which compares, for example, to 440 kg in Argentina (22). These dramatic and difficult transitions have dislocated herds, particularly with sell offs from large state enterprises to individuals and families. One consequence is a general lack of awareness about the immune status of animals on part of livestock owners and veterinary surgeons (4).

The disease agents as described present challenges to Kazakhstani development strategies targeting beef, given that the Sanitary and Phytosanitary (SPS) guidelines promulgated by the Office International des Epizooties (OIE; now the World Organization for Animal Health), which at the present time would probably disallow export to the markets within its purview. Moreover, export itself brings risk, given outbreaks of FMD and Brucellosis can prove costly. The 2001 FMD outbreak in the UK provides a case in point. The epidemic, which began on a pig farm in February, 2001, spread to at least 40 other properties in just a few weeks. By the end of September, after which no more outbreaks occurred, FMD had been reported on 2026 properties. Culling took place on these, neighboring, and proximity properties, totaling to ~8131. Altogether 4 million animals were slaughtered to control the outbreak, with an additional 2.5 million on “welfare grounds,” numbers large enough to obscure any estimate of prevalence (23–26). By the time, the UK was declared FMD-free and cleared for export in January 2002, the disease had cost ~9 billion $US for culling, emergency vaccinations, and intensified surveillance (3, 27). Losses due to trade, pursuant to OIE and World Trade Organization (WTO) regulations, also added to the ledger. The UK FMD outbreak provides a hard lesson for all countries seeking to build export earnings through the trade of animal products.

Although a land-locked nation, Kazakhstan is surrounded by large markets in China (population 1.4 billion) and Russia (population 143 million) where climatic conditions significantly constrain animal husbandry. Adding to this and still within reach are the desert countries of the Middle East, and the densely settled lands of the European Union. Kazakhstan’s Ministry of Agriculture is currently aiming to increase exports by orders of magnitude in no more than 5 years, from today’s 1,000 metric tons to 180,000 by 2020 (2). Pursuant to this development objective, Kazakhstan has initiated herd improvements with the purchase of Angus and Hereford cattle from the US, Canada, and Australia, in an effort to raise the genetic qualities of its resident animals (2). But translating genetic quality into a robust export sector will require a number of additional investments, as, for example, in sanitary infrastructure capable of maintaining OIE standards for international trade in food products. As a member of both the Customs Union of the former Soviet Block, and the WTO, Kazakhstan has committed itself to such improvements with sizeable budget allocations (28). Although some importers are willing to forgo OIE’s stamp of approval, it is within Kazakhstan’s long-run interests to build healthy herds of cattle and small stock.

## The South American Experience

Improved animal sanitation has proven economically important throughout the world, and contributed substantially to the development of countries with comparative advantage in land resources. This is especially true for South America, where the agricultural sector has often functioned as an engine of growth. With respect to livestock and meat products, Brazil and Argentina come immediately to mind as countries that improved their economic well-being by promoting international trade among their producers. Although Argentina has deemphasized livestock herding in favor of field crops like soybeans over the past decade, ranching long generated considerable export earnings (29). For its part, Brazil has joined the world stage as a powerful BRIC country, despite its current economic difficulties. This transformation has been partly enabled by agriculture, which includes management of the world’s largest cattle herd, and position number two as a global exporter of beef (30). Brazil’s ascension as a globally significant beef exporter has taken about a decade, given no Brazilian state was declared FMD-free until 1998.

Thus, South American countries potentially provide lessons in the leveraging of economic development outcomes from strategic interventions by federal governments in animal husbandry, and by engagement in export markets more generally. This is accentuated for Kazakhstan by similarities along physical, social, and economic dimensions, particularly with respect to Brazil. Both Brazil and Kazakhstan are large countries, possessing abundant land resources, with Brazil covering 8,515,767 km^2^. Low levels of population density and the persistence of natural environments throughout South America means that Brazil retains within its boundaries ecological reservoirs of the FMD and Brucellosis disease organisms, as is the case in Kazakhstan (31, 32). As for levels of economic development, Brazil and Kazakhstan are also similar, with per-capita GDP reaching 11,690 US$ in Brazil, comparable to that of Kazakhstan (see above). We now consider efforts to control and eradicate FMD in Brazil, which spanned much of the twentieth century and involved a long-term process of policy adaptation. Although not discussed explicitly, the lessons learned in controlling FMD apply to Brucellosis and other biothreats.

### Combatting FMD in Brazil

The relocation diffusion of FMD from Europe to South America occurred in the late nineteenth century, infecting herds in Brazil, Argentina, and Uruguay (33). Nevertheless, Brazil did not grow serious about solving the problem until the founding, in 1951, of the Pan-American Center of FMD (PANAFTOSA) in Rio de Janeiro. An initial strategy focused on prevention of the disease altogether, as Brazil (with help from the Word Health Organization) developed an FMD vaccine and provided credit lines to ranchers for implementing sanitary procedures (34). Despite these various initiatives, continuous outbreaks of FMD eroded both South American and Brazilian dreams of a continental export economy based on beef. An awareness that its FMD policies were not working, together with a growing interest in export on part of the private sector, inspired a significant policy shift, not only in Brazil but also throughout the continent. Consequently, the countries of South America agreed to the 1987 Hemispheric Plan for the Eradication of FMD, or PNEHA (35). This plan pursued a vigorous vaccination campaign of continental proportions, and declared a 95% coverage for the South American herd by 1995. The veterinary strategy of PNEHA addressed the disease epidemiology of cattle systems, with a focus on FMD-endemic areas and on the spatial links between grazing ranges and fattening operations (33). PNEHA responded immediately to outbreaks by controlling animal movements between affected and unaffected areas, and by mass vaccinations of susceptible animals; in areas disease-free before an outbreak, PNEHA recommended culling of affected and exposed animals (36).

Brazil’s implementation of PNEHA via its Ministry of Agriculture in 1992 soon began to bear fruit, with a multi-pronged approach involving vaccination campaigns, capacity building of dedicated bureaucracies (e.g., Department of Animal Health), and the control of animal movements. The OIE had by then set international sanitary standards for the trade of animal products, creating strong incentives for exporters to improve livestock health. In addition and fortuitously for Brazil, OIE relaxed its requirement that countries be entirely free of FMD before engaging in trade. Starting in 1992, beef and beef products could originate from areas within a country certified to be FMD-free, even if the disease existed elsewhere inside national borders. The Brazilian approach aimed to eradicate FMD by regionalizing and decentralizing its efforts, and by stimulating the formation of public and private partnerships (37).

The regionalization, keyed to OIE’s new spatial sensitivity to the environmental circumstances of animal husbandry, partitioned Brazil into five territorial extents, or *circuits*, to be managed individually in the fight against FMD, and which could then source export goods once FMD had been internally controlled (38). PNEFA also promoted decentralization by transferring programmatic responsibilities to Brazil’s 26 states, and by direct involvement of civil society through outreach to producer associations. Ultimately, PNEHA implemented an effective division of responsibilities across both civil society and government (37). At the federal level, the Ministry of Agriculture acted as overall program manager and credit source, while individual states provided front-line veterinary services. For its part, the private sector managed culling during outbreaks, and created an emergency fund for vaccination and for the financial strain of monetary losses (37).

**Figure 2:**
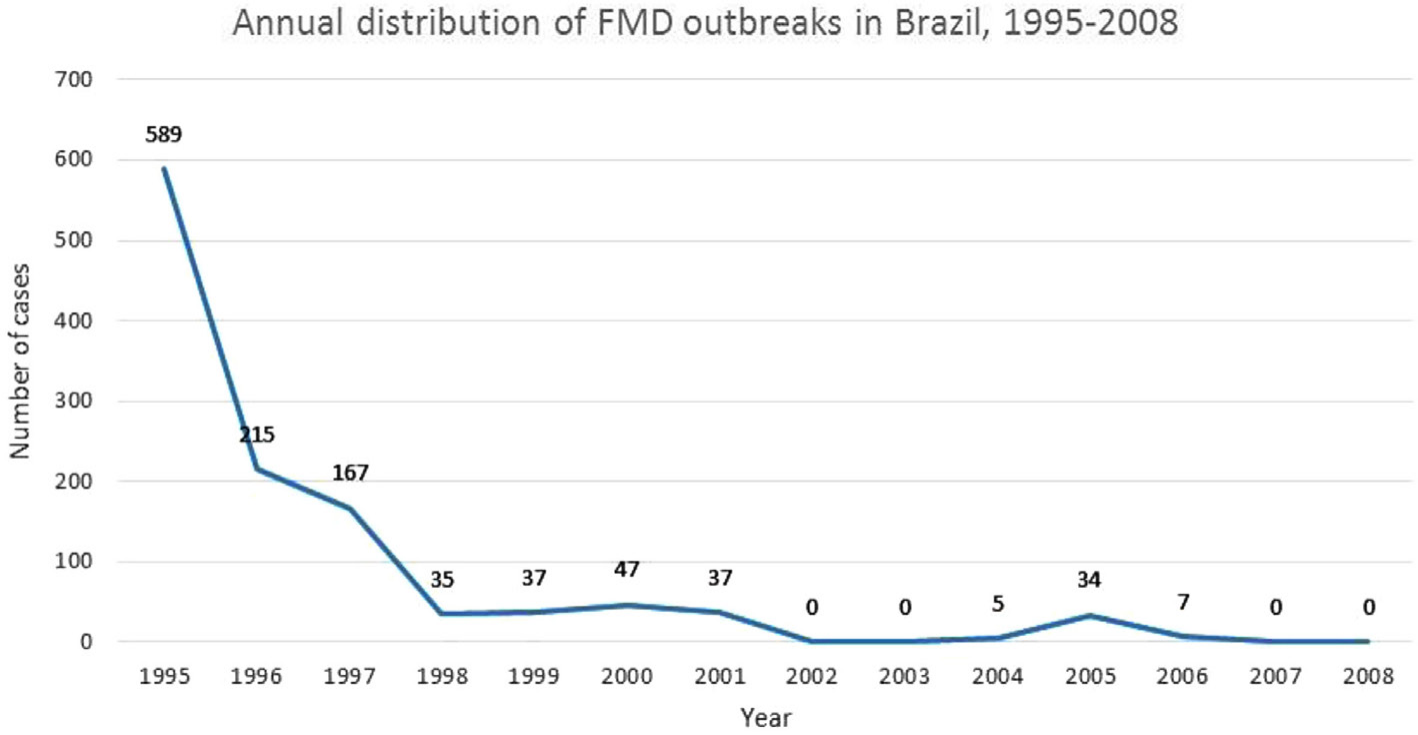
**Annual distribution of FMD outbreaks in Brazil, 1995–2008**. Adapted from Departamento de Saude Animal (40).

Figure 2 depicts PNEFA’s dramatic impact on the FMD status of the Brazilian herd. The reported number of outbreaks is 589 in 1995, after which it drops precipitously, falling by an order of magnitude through the late 1990s. By 2002, outbreaks reach 0, with an uptick through 2006, after which the number returns to 0. The decisive drop appears to occur in the 1990s, in correspondence with Brazil’s policy shift (39). FMD control manifests a distinct spatial pattern for the Brazilian case, with a move from southern to northern latitudes. Before 2000, only two southern states (Rio Grande do Sul and Santa Catarina) enjoyed OIE certification, but by 2004 the situation had changed dramatically, with most of southern and central Brazil engaged in export, even the Amazonian states of Acre, Rondonia, Mato Grosso, and Tocantins. OIE-certified areas regressed in 2005, after FMD outbreaks in Mato Grosso do Sul, Parana, Mato Grosso, and Tocantins. In all likelihood, the problem began with initial infections in Mato Grosso do Sul, a lightly settled area near the Paraguayan border, where vaccinations were not always effectively administered. In any event, the outbreak areas were sanitized by 2008, and new lands, cleared for export, including a large part of the Amazonian State, Para (40).

### FMD Policy Successes and Failures

Despite the dramatic drop in outbreaks depicted by Figure 2, Brazil’s success in controlling FMD was slow in coming. There is little doubt that PNEFA played a decisive role in ultimately reducing FMD outbreaks, and features of the program (e.g., vaccination coverage and sanitary structures) have been shown to associate strongly with FMD control (37). That said, other factors outside the realm of policy intervention have played an important role in Brazil’s experience with FMD, particularly in sustaining its long-run persistence. One of the early difficulties in sparking private sector commitment resided in a set of perverse incentives faced by large-scale ranchers. Given their early markets were primarily domestic, they saw little advantage in undertaking sanitary improvements vis-à-vis FMD, given costs associated with assembling large herds for regular vaccinations outweighed the potential losses from an FMD outbreak. A second reason that FMD control efforts remained ineffective through much of the twentieth century can be attributed to social and political turmoil, and associated economic difficulties such as inflation and poorly functioning credit markets. Only with currency reform under the *Plano Real* in the early 1990s did the macro-economy stabilize sufficiently to engender a robust, agricultural expansion (39). Yet another reason that FMD proved resistant despite substantial initial efforts at control can be found in the wider experience of the South American continent, with its history of *caudilismo*, autocratic rulers, and corruption, all of which engendered a generalized distrust of government institutions. In the Brazilian case, this meant ranchers tended to avoid front-line organizations, such as the Veterinary Sanitary Service, a response that would stymie even the best-laid plans (34). The return to democracy in 1985 paved the way for new attitudes on part of the private sector and the population as a whole.

## Computational Approaches

The Brazilian case provides useful lessons for Kazakhstan, and Central Asia more generally, about how to combat FMD and Brucellosis in the interest of agricultural development. Thus, Kazakhstan is in the enviable position of being able to learn from someone else’s successes, and failures. But that is not all, as Kazakhstani planners also have the advantage of being able to draw from a wide range of newly available computational approaches to epidemiology, with the potential to facilitate both the design and implementation of policy. Geospatial analysis, for example, has provided critical insight into the formation of animal disease clusters, and yielded descriptive and inferential assessments via spatial modeling (41). Clustering analyses comprise statistical approaches that test whether or not outbreak events are more spatially proximate than would be expected under randomized processes. Spatio-temporal analyses, such as accomplished by implementing a spatial scan statistic [see below; (42, 43)], evaluate if outbreaks (or other phenomena) cluster in both space and time, thereby shedding light on why clusters form, their size, and their duration. For the purposes of this article, we define spatial models as techniques that predict the spatial distribution of disease risk, or explain the spatio-temporal trends of outbreaks.

### Clustering

AlKhamis et al. (44) applied a *direction test* within space-time clusters – defined using spatial scan statistics – to describe FMD transmission patterns across Israel in terms of size (square kilometer) and seasonality of outbreak clusters, and infection spread directions. Spatial scan statistics search for the most likely cluster of cases by placing varying sized circles over case locations and comparing disease rates within and without the circles; circle sizes are varied up to the distance necessary for reaching a user defined maximum proportion of the population at risk. The temporal element is incorporated by a cylinder, with height measured in units of time (45), while directionality of cluster spread is established by a direction test, a two-dimensional mapping that calculates the average direction in which cases move, with significance computed by Monte Carlo simulation (46). Using an approach similar to AlKhamis et al. (44), Schlak (10) applied the direction test method to evaluate seasonal spread of FMD across the southernmost oblasts of Kazakhstan and showed significant linkage between outbreaks associated with seasonal peaks for the period 1955–1964. A study in Mongolia on Kazakhstan’s eastern border employed the spatial scan statistic and the direction test to FMD data, and illustrated the direction in which outbreaks moved (47). Recently, Sytnik et al. (11) has applied kernel density estimation (KDE) to categorize risk nationally and has identified disease hotspots in southern Kazakhstan for the period 2011–2102. These recent hotspots tend to overlap with those identified by Schlak (10) for the period 1955–1964, over 60 years ago, suggesting outbreak areas of surprising persistence. Southern Kazakhstan borders Uzbekistan and Kyrgyzstan, each with a history of FMD; this highlights the trans-boundary nature of the problem for central Asia as a whole (48, 49).

### Spatial Modeling

A growing number of studies apply spatial models to examine or predict the spread of animal diseases in a variety of geographic settings. For example, Lawson and Zhou (50) applied a Bayesian framework in evaluating biweekly count data at farm-scale to examine the 2001 UK FMD epidemic, a study that details the effects of disease control via vaccination and culling. Ward et al. (51, 52) employed geospatial simulation models to examine the role of wildlife in initiating livestock outbreaks in Texas. They showed that feral hogs (*Sus scrofa*) and white-tailed deer (*Odocoileus virginianus*) precipitated different cattle outbreak patterns, thereby providing insight how wildlife populations function as disease reservoirs, and how species spillovers can initiate livestock epidemics. The Texas study is particularly relevant to Kazakhstan, where *Saiga* antelope (*Saiga tatarica*) are highly sensitive to FMD and probably function as reservoirs for the disease. Using a spatio-temporal model, Morgan et al. (32) showed that virus spillover in either direction between antelope and livestock depends on *Saiga* migration timing and herd size. *Saiga* peak infection is greatest in spring and autumn, when calving and maternal immunity wear off, respectively. In light of the historical work by Schlak (10), FMD rates in cattle appear highest in southern Kazakhstan during the late summer and autumn months, a period that overlaps with *Saiga* spillover risk.

## Discussion

Computational modeling and analysis promise to help countries like Kazakhstan gain the epidemiological insight needed for controlling and eradicating biothreats, such as FMD and Brucellosis. As noted at the outset, Kazakhstan has taken a development path intent on using its pastoral resources to maximum extent by becoming a major beef exporter. This will require the shaping of relevant sanitary policy. The case study of Brazil indicates a broad set of factors that ultimately helped achieve control of a key biothreat, FMD, in pursuit of development objectives. Some of these reach far beyond responses based on the veterinary and epidemiological sciences, like the degree of societal trust in government institutions. For the Kazakhstani case, the transition to a market economy has been difficult overall, and many problems remain on the agricultural front, such as how to redistribute land and incentivize producers after ~60 years under a socialist government (20). While we appreciate the importance played by cultural and social context, we limit our remarks here to the technical side of the issue relating to veterinary policy vis-à-vis biothreats to livestock, and to the role of computational science in policy formulation. We do not consider important animal husbandry issues relating to herd structure (beef vs. dairy) and slaughter weight (22).

With a cattle herd of ~200,000,000 animals, distributed across ~2,700,000 rural properties, Brazil presents a challenging epidemiological case for reasons of sheer size. Adding to this are the disease vectors of the wild animal carriers found in its expansive, ecologically intact regions (31). Despite these daunting circumstances, the Brazilian government managed to control its FMD biothreat in only a few years, although success was long in the making. The PNEHA policy relied on a social compact involving state decentralization and public–private partnership. That said, the best administrative intentions make little headway without the commitment of financial resources. In the Brazilian case, these were substantial. PNEHA programmatic costs start out at about 100,000,000 $US in the early 1990s, and by 2008 they climb to nearly 450,000,000 $US, or about 2 $US per animal. These funds were spread across a large number of expenditure categories, including thousands of physical structures (e.g., surveillance posts along highways) and the creation of a dedicated labor force of ~2,500 veterinarians, together with support staff, both technical and administrative (40).

Political and social adjustments following the post-Soviet period have not been easy for Kazakhstan, and its economy continues to evolve with ongoing institutional reform. Nevertheless, with its herd of 6 million cattle, the biothreat to Kazakhstani livestock seems small in comparison to what Brazil faced only a few decades ago, at least from a numerical perspective. As already mentioned, Kazakhstan wishes to boost its current 1,000 metric tons of beef export to 180,000 by 2020 (2, 22). Given only 35% of existing pastures and hayfields are used, or about 630,000 km^2^ out of 1,820,000 km^2^, such an expansion would seem reasonable for a production system based on rangeland grazing (22). The export production target would generate nearly a billion dollars (720,000,000 $US) at current international beef prices (53). If we assume annual costs of FMD protection in Brazil at ~2 $US per animal, a herd on the order of 20,000,000 animals generates potential earnings far in excess of costs associated with OIE’s SPS export requirements (54). Given Kazakhstan’s willingness in advance to use earnings from its mineral and fuel exports to promote economic development in other sectors, the control of FMD as well as Brucellosis would appear to be within financial reach.

The preceding section suggests that geospatial technologies could be effectively implemented to help Kazakhstan best develop its pastoral resources. Such computational techniques were in their infancy when Brazil began concerted efforts to manage its FMD biothreat situation, so there is no prior experience to draw from in this regard. Nevertheless, from the applications to date, we argue that the right combination of spatio-temporal analyses – such as the direction test (44) and predictive modeling such as with “random forests” (55) – make possible the design of a spatially sensitized approach to the distribution of veterinary resources, one that could minimize costs and maximize revenues in terms of animal health and well-being. Although much of the computational research addresses FMD, geospatial program design would apply with equal force to other worrisome biothreats such as Brucellosis. Specific veterinary applications in the Kazakhstani case could:

1. Identify hotspots of disease clustering, in the interest of optimal spatial allocations of human resources for disease prevention and control. Similarly, identify “coldspots” to understand disease suppression mechanisms and environmental factors not conducive to FMD or Brucellosis infection. Use cluster sizes to define buffer zones within which to apply control measures during outbreak events (44).
2. Shape biodiversity configurations of the Kazakhstani landscape in order to ensure healthy domestic livestock and wildlife herds, given both populations may harbor diseases, with prevention less tenable in free ranging wildlife like the *Saiga* antelope. Identify natural buffers to disease transmission, and the optimal placement of fencing and other physical impediments to animal mobility.
3. Determine zones of disease likelihood in the interest of partitioning Kazakhstan into *circuits* with variable degrees of risk, as was done in Brazil, given OIE’s acceptance of export from countries not entirely FMD-free. Expand from KDE to a probability-based prediction of disease risk (11).
4. Find conduits of disease transmission at region-scale and, together with geospatial information on transportation systems, isolate target points for the optimal placement of control structures. Use direction tests to assess needs for border control (44).
5. Analyze spatio-temporal outbreak patterns to develop a cost-minimal program of vaccination, including seasonal timing of vaccines (44).

We have focused our discussion on Kazakhstan as a site for the potential development of computational approaches to biothreat control. That said, it is important to note that the entire central Asian region would benefit both from an improved veterinary situation in Kazakhstan and from policy formulation that exploits the new powers of geospatial technology.

## Conclusion

Biothreats continue to put livestock herds at risk worldwide, a circumstance with enormous implications for human welfare. Nowhere is this empirical observation more in evidence than for central Asia, where FMD and Brucellosis have long been endemic. For countries like Kazakhstan that once belonged to the FSU, institutional adjustments following its collapse have proved difficult, and presented challenges along a variety of fronts like that of building an economy that best serves its citizens. Kazakhstan, with an abundance of rangelands and deep cultural traditions of animal husbandry, seeks to secure its place in the global economy by improving opportunities for the export of animal products, notably beef. This reflects a perspicacious assessment of economic potential, but it will not be successful without a concerted effort to raise the health conditions of its livestock herds. The global economy provides opportunities for trade and foreign exchange earnings, but it is also a demanding task-master that places stringent demands on product safety relative to SPS standards.

The demands of the global economy can be met, however, as the case of Brazil testifies. Here, outbreaks of FMD fell precipitously in only a few years, once the Brazilian government pursued a public–private partnership that enabled the efficient decentralization of policy implementation. The loosening of OIE requirements – allowing for export from FMD-cleared zones within a country even if the biothreat persists elsewhere – dovetailed with Brazil’s spatial approach to divide and conquer by partitioning its control efforts into *circuits*. Brazil’s policy approach was not a silver bullet, given the importance of both institutional and economic change that ultimately provided the set of necessary background conditions enabling success. The parallels with Kazakhstan in this regard should provide grounds for encouragement, though. As Brazil moved away from a development model that inhibited market forces, and built new trust with its citizenry, its veterinary programs proved successful in a short period of time, enabling quick market consolidation and its number 2 rank as a global beef exporter. Kazakhstan presents a more difficult epidemiological case than Brazil given that policy to improve livestock health will have to fully integrate the wildlife factor, given conservationist concerns for the *Saiga* antelope, which may serve as livestock reinfection reservoirs. Luckily, the rapid evolution of geospatial technology provides a powerful new toolkit that can help governments like Kazakhstan bring development benefits to their peoples, in the face of analytical challenges to the design of policy.

## Conflict of Interest Statement

The authors declare that the research was conducted in the absence of any commercial or financial relationships that could be construed as a potential conflict of interest.
